# Supplementary material for: Osteopontin Deficiency Ameliorates Prostatic Fibrosis and Inflammation
Source: Int J Mol Sci. 2021 Nov 18;22(22):12461. doi: 10.3390/ijms222212461 (PMC8617904; doi:10.3390/ijms222212461)
Supplement: Supplementary file 1 [file ijms-22-12461-s001.zip › supplementary figures.pdf]

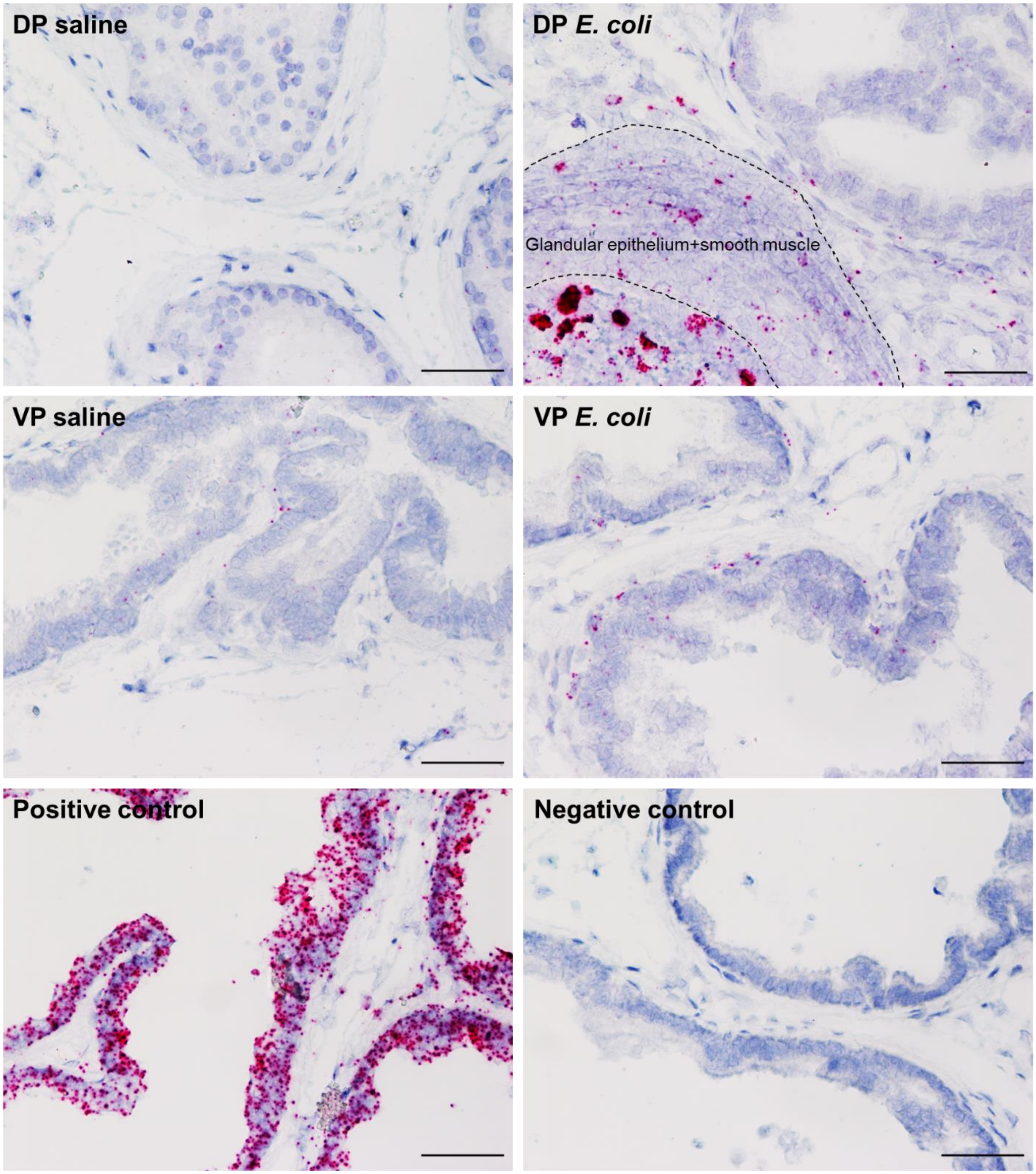

Supplemental Figure S1 *Spp1* expression is upregulated in the dorsal prostate (DP) in *E. coli*-instilled mice. *Spp1* expression appears to be upregulated in multilayered prostate ducts as well as in the lumens that are occupied by immune cells. The ventral prostate (VP) only contained sporadic upregulation of *Spp1* expression. The positive control probe was provided by Advanced Cell Diagnostics and targeted a gene that has medium expression level across all cells. For the negative control, no probe was added. Scale represents 100 µm.

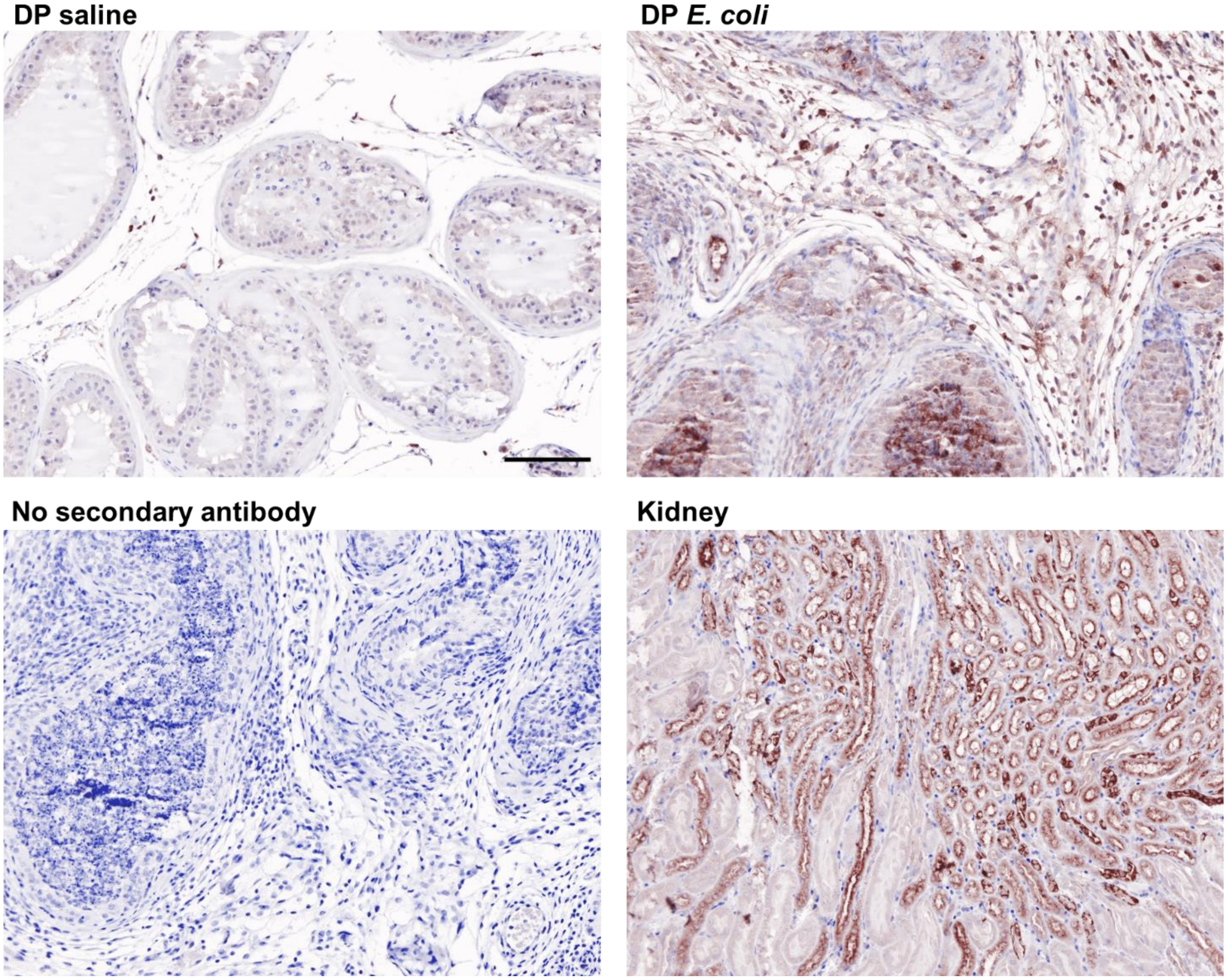

Supplemental Figure S2 Osteopontin (OPN) protein expression is upregulated in the dorsal prostate (DP) in *E. coli*-instilled mice. OPN expression appears to be upregulated in multilayered prostate ducts as well as in the lumens that are occupied by immune cells. Images were captured at 20x magnification. Scale represents 100 µm.

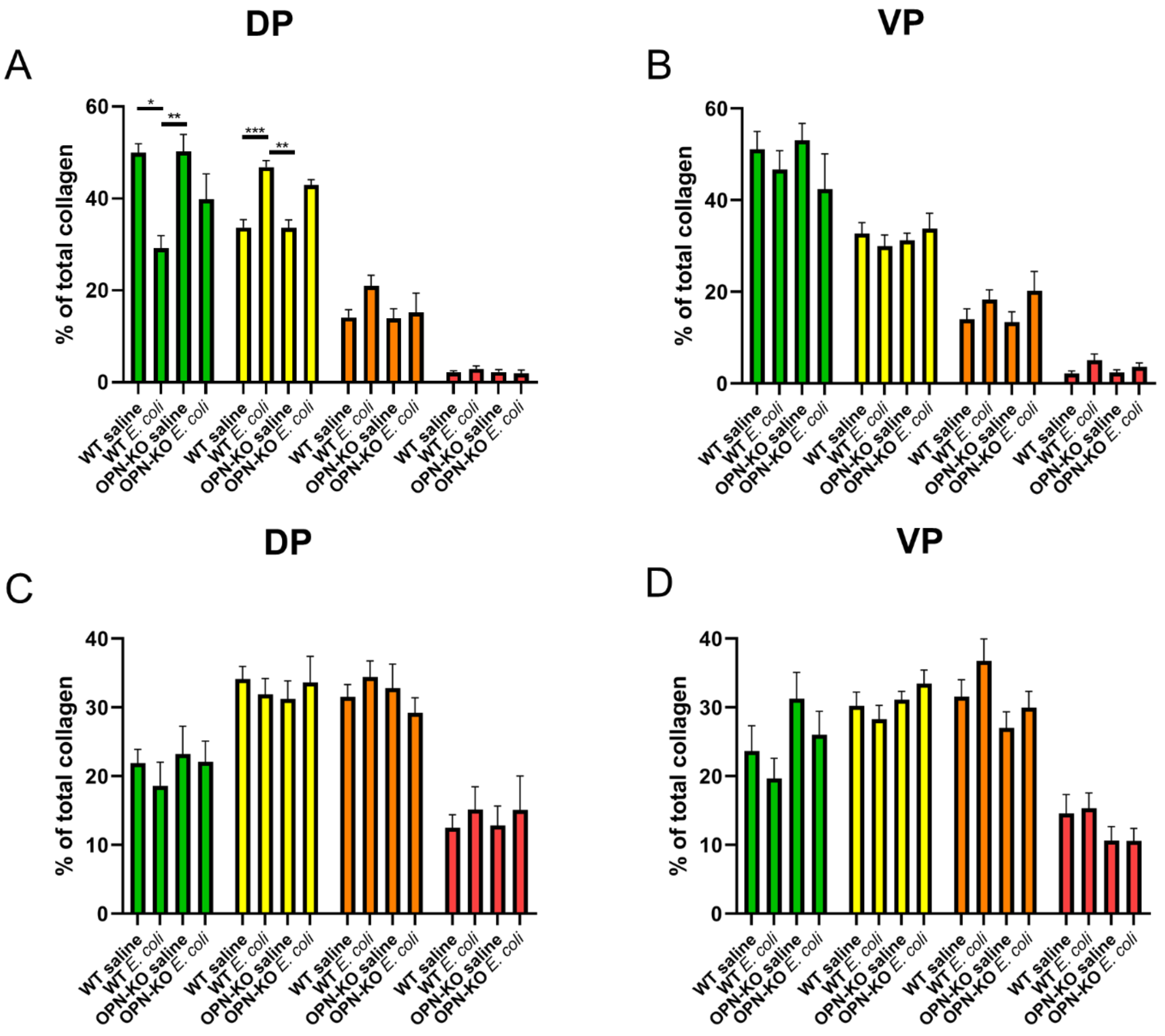

Supplemental Figure S3 The distribution of collagen polarization colors change similarly in wild type (WT) and osteopontin knockout (OPN-KO) *E. coli*-instilled dorsal prostates (DP). One week after the bacterial instillation (A and B), we found significant decrease in green color and increase in the proportion of yellow fibers in the DP, but not in the ventral prostate (VP), in WT mice which indicates an increase in collagen thickness. There was no significant alteration in the representation of polarization colors after two months (C and D). Tissues were stained with PSR and images were taken with a circularly polarized filter. Significance was determined by the Kruskal-Wallis non-parametric test. *: $p<0.05$; **: $p<0.01$; ***: $p<0.001$.

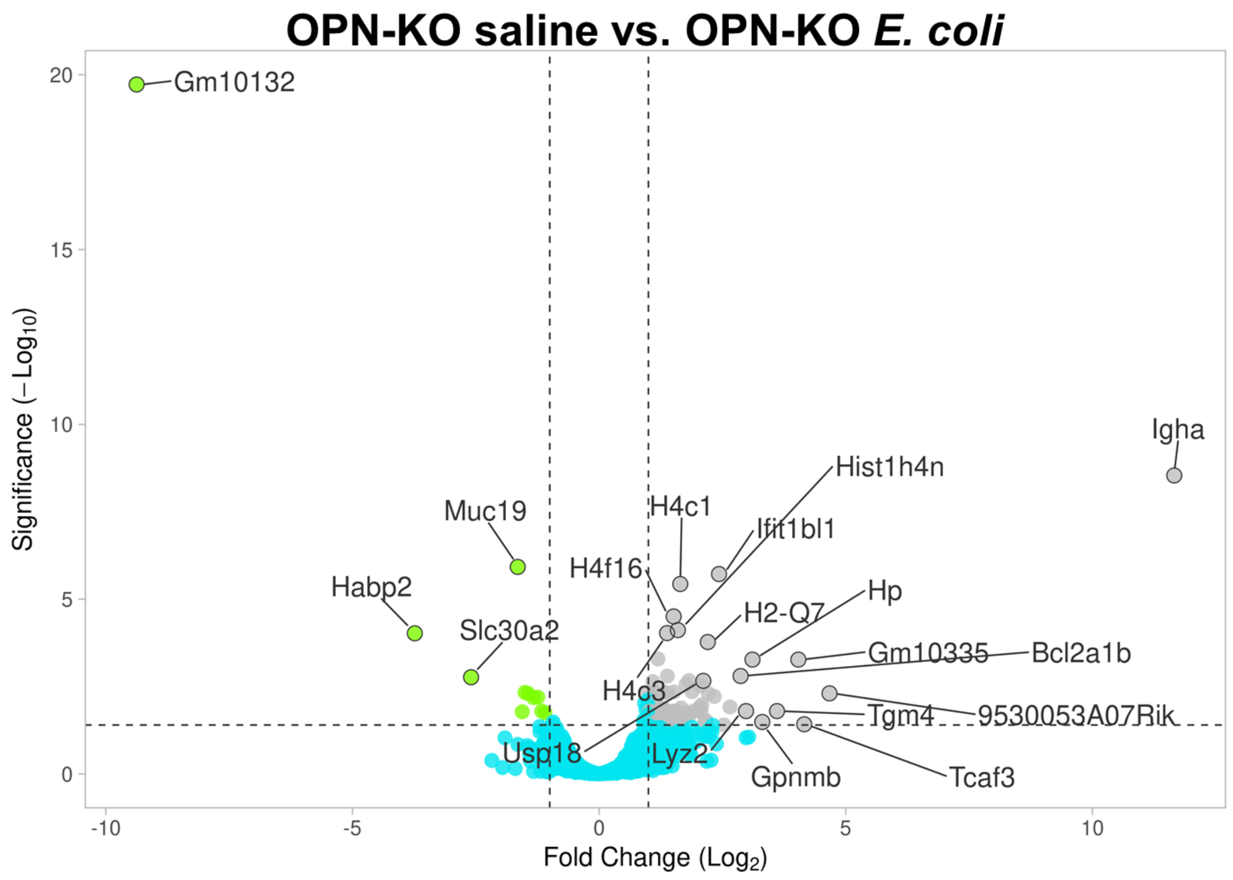

Supplemental Figure S4. Osteopontin (OPN) deficiency prevents *E. coli*-induced expressional changes in genes associated with inflammation and fibrosis. The 20 top hits determined by Manhattan distance with the VolcaNoseR application are labelled.

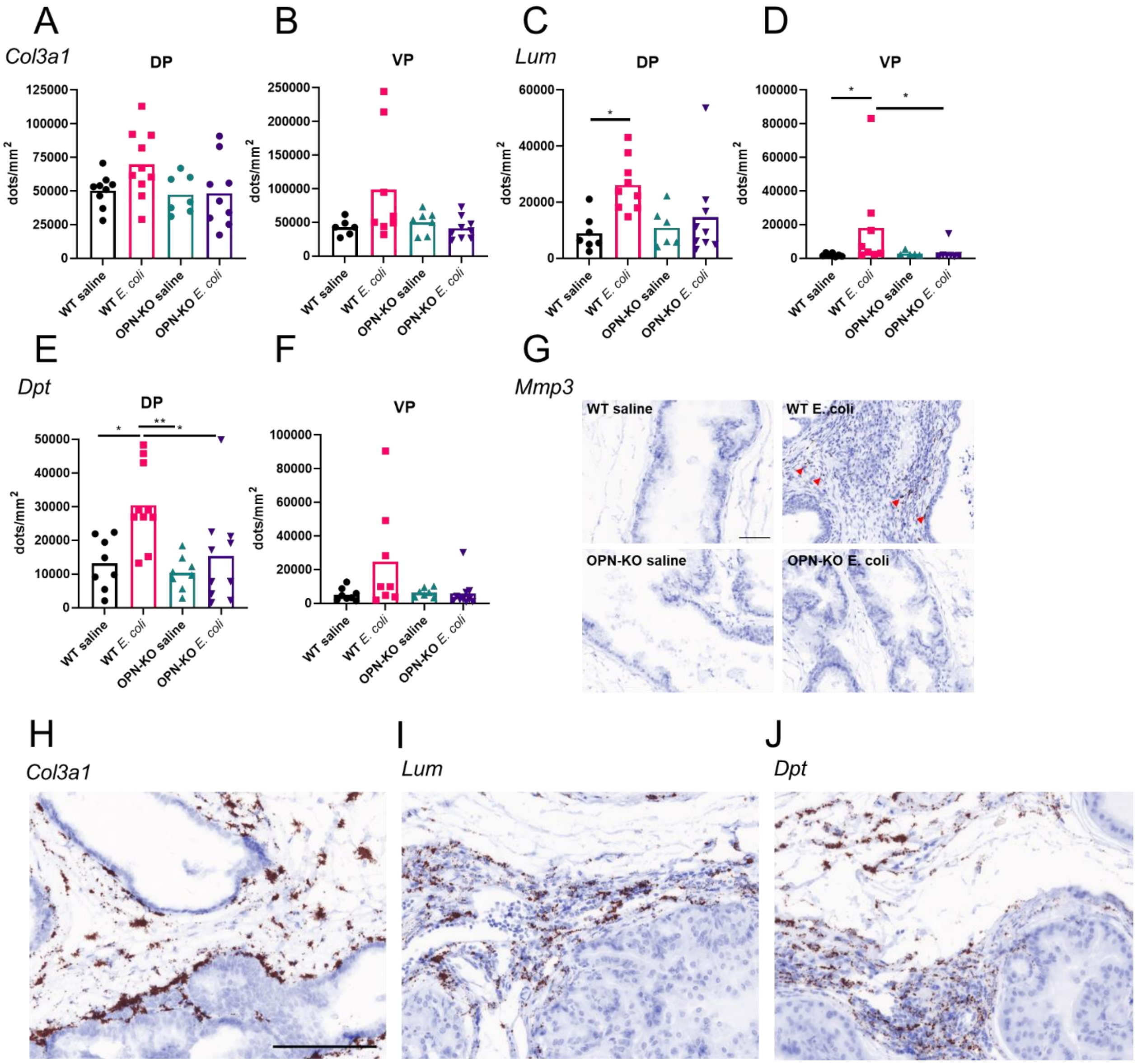

Supplemental Figure S5. RNAscope analysis of pro-fibrotic genes, *Col3a1* (Panels **A** and **B**), *Lum* (Panels **C** and **D**), *Dpt* (Panels **E** and **F**) and *Mmp3* (Panel **G**) in the dorsal (DP) and the ventral prostate (VP) across all experimental groups. Quantification of mRNA abundance for *Col3a1*, *Lum* and *Dpt* was performed using the Mantra 2 Quantitative Pathology Workstation. Due to the limited expression of *Mmp3* in uninflamed tissues, representative images are shown instead of quantification. Representative images for *Col3a1*, *Lum* and *Dpt* are shown in Panels **H**, **I** and **J**, respectively. Significance was determined by the Kruskal-Wallis non-parametric test. Images were captured at 40x magnification. Scale represents 100 µm. *: $p<0.05$; **: $p<0.01$.
