# Supplementary material for: Osteopontin Deficiency Ameliorates Prostatic Fibrosis and Inflammation
Source: Int J Mol Sci. 2021 Nov 18;22(22):12461. doi: 10.3390/ijms222212461 (PMC8617904; doi:10.3390/ijms222212461)
Supplement: Supplementary file 1 [file ijms-22-12461-s001.zip › supplementary legends.pdf]

Table S1: Analysis of differently expressed genes in the WT saline vs. WT *E. coli* contrast.

Table S2: Analysis of differently expressed genes in OPN-KO *E. coli* vs. OPN-KO saline contrast. LogFC values are also shown for the reverse contrast (OPN-KO control vs. OPN-KO E. coli) that were used to create Figure S4.

Table S3: Analysis of differently expressed genes in the vs. WT *E. coli* contrast. LogFC values are also shown for the reverse contrast (WT E. coli contrast vs. OPN-KO *E. coli*) that were used to create Figure 7E.
